# Practical Medicine

**Published:** 1876-09

**Authors:** 


[Superabundance of "copy" has compelled the editors to allow but little space to this department this month. The intention has been, and still is, to make the Summary of Progress, which is the result of much labor, one of the most prominent features of this Journal.]

### I. Practical Medicine.

1. *Cure of Tetanus by Mechanical Measures.* Calastrie. (*Gazaetta Medic*, Lombardia, No. 27.)

A patient, who was convalescent after various attacks of hæmorrhage from the skin and mucous membranes, was wounded in the sole of the right foot and had partial tetanus result. There was considerable rigidity of the muscles of the cervical region and those presiding over the movements of the jaw. Sulphate of quinia and chloral were followed by temporary relief only. Then C. thinking that the difficulty of injecting sufficient nutriment was due to the localization of the tetanic affections in the muscles named above, concluded to overcome the rigidity by forced movements of flexion, extension and rotation. This was continued till voluntary movement became possible. The jaws were then separated little by little from day to day, until they could be more and more widely opened, and the patient could himself take solid food. This treatment continued during the month of August, and by autumn the cure was completed.

2. *Case in which foreign bodies were inserted in the brain with suicidal intent, and retained several months.* W. B. Carpenter, M.D. (*Am. Jour. Med. Sci.*, April, 1876.)

The patient was a State prison convict, twenty-eight years old, who was insane. He was a strong, healthy man, with a well developed brain, weighing 56 ounces. Two years before his attempted suicide he had been discharged from an insane asylum where he had spent a year. During the time subsequent to this discharge he was kept in the hospital of the prison. He was caught one night stuffing No. 20 broom wire into the cavity of his cranium through an opening in the skull he had made, about an inch above the right ear with an awl. Over four inches of the wire was withdrawn. A month later he drove an awl into his brain through the skull near the top of the head. Soon afterward he was found thrusting another piece of wire laterally through his skull, and it is known the piece reached through to the opposite side of the brain. From this there only resulted slight paralysis, which in two weeks was nearly well. His desire to die now ceased. Months afterward he, as it was believed, by pure accident, took an overdose of morphine to procure sleep, and died.

*Post mortem.* There were found in the right hemisphere, some of them resting on the dura mater at the base of the brain, the following articles: Two pieces of wire, each over two inches long; a four-penny nail, 2¼ inches long, and a needle 1⅝ inches long. Two of these bodies were found in the middle and two in the antérior lobe. All "were more or less insulated by a gelatinous substance closely adhering to them." There had never been a loss of any of the special senses, or any symptoms of aphasia.

3. *Gangrene of the Limbs in Typhoid Fever.* VALETTE. (*Lyon Médical*, Feb. 6, 1876.)

The author gives an interesting account of a young servant girl, attacked with typhoid fever and pneumonia of one lobe of the right lung, whose condition became so deplorable that large purulent ulcerations occurred over the trochanters and sacrum, extensive portions of the skin mortified over the perineum and vulva, and two-thirds of one foot became black and mummified, with a surrounding line of demarcation. Amputation was performed at the urgent request of the sufferer, to relieve her of her pain, which no form of narcotic dressing could assuage. She survived twenty-three days.

*Post mortem.* About 25 cicatrices were found in the small intestines, red heptization of the inferior lobe of the right lung, left ventricular heart clot, and a *whitish gray clot at the point of bifurcation of the primitive iliac artery, closely adherent to the vascular walls, its superior half free and closing like a valve the external iliac artery.*

The author concludes: 1st. That gangrene of a limb may be classed among the possible complications of typhoid fever. 2d. That the gangrene is not to be attributed to that ill defined and almost unintelligible cause known as malignity, but to determinable organic conditions. 3d. That this complication may be due to arteritis, developed in a manner similar to that of other causes in typhoid fever. (Patry.) But as shown in this case, the gangrene may be determined by an embolism, as described by Bonnet, Van Swieten and Virchow.

4. *Diapedesis of Leucocytes and its present pathological significance.* DR. N. B. SIGER. (*Proceedings Med. Society, County of Kings*, June, 1876.)

After a review of the history of the studies on this subject and a reference to the different experiments for the demonstration of the processes of inflammation and the behavior of leucocytes in this and other pathological conditions, he sums up from the discussions of the subject the present state of the case, as follows:

1st. The greater part of all pus, wherever found in the body, either in vascular or non-vascular tissues, is made up of emigrated white blood cells or their descendants.

2d. Whether the normal cells of the part proliferate in inflammation is at present *sub judice;* the preponderance of evidence is against it.

3d. While emigrating, the leucocytes rapidly proliferate in the tissues.

4th. The emigrated cells are capable of undergoing metamorphosis, and of being transformed into fixed elements, similar to those of the tissue in which they are situated; this is shown in the healing of wounds, where the granulating tissue, which is now known to be made up of emigrated cells and their descendants, is transformed by contact with the normal parts into connective tissue and epithelial cells.

5th. Hence, organization is accomplished by the fixation of the wandering cells and their transformation into cells like those of the neighboring tissues.

6th. When suppuration occurs in mucous or serous surfaces, while most of the pus is composed of leucocytes, the participation of the local epithelium in the process is extremely probable.

7th. The youngest epithelial cells of a tissue are in all probability nothing but organized wandering cells.

8th. Diapedesis is believed to be as prominent a phenomenon in the so-called chronic forms, as in ordinary acute inflammations.

9th. Morbid growths generally, which do not belong to the connective tissue type, are believed by many to be intimately connected with the emigration of cells at an irritated point.

10th. The recurrence of malignant growths after careful extirpation, is believed to be due to the emigration of the young cells of the tumor, which possess an undoubted power of amœboid motion.

5. *Profuse Salivary Flow and Swelling of the Tongue.* W. CARSON, M.D. (*Cin. Lancet and Observer*, June, 1876.)

The patient was a woman of fifty; she had had for some years cardiac disease of some sort; she had pneumonia involving one lung, then as this was recovering she had supervene decided symptoms of meningitis—delirious actions and occasionally drowsy periods—when it was found she had a large quantity of albumen in her urine. Now, one morning when the tongue was dry and small and smooth it suddenly became moist, large and swollen, so that it filled the entire space between the teeth. One-half the tongue was more swollen than the other, and there was an enormous out-flow of apparently salivary and viscid fluid, without coughing or vomiting. Otherwise there was no change in the patient's condition, and these conditions continued until the death of the patient. How long a time this was, we are not informed. No mercury had been given. Dr. C. refers to the various experiments that have demonstrated the connection of the chorda tympani nerve, the glasso-pharyngeal and the sympathetic with the circulation in the tongue and salivary glands, and then says: "It would seem then, that the principal medium, by which this association of congestion or tumefaction of the tongue and profuse salivary flow is produced, is the chorda tympani nerve, with some added effect from the vaso-dilating fibres of the glasso-pharyngeal." Again he says: "The facts are of great physiological importance, as proving the existence of active vascular dilation, under stimulation of a few nerves; but they have beside, their clinical aspect." "The experimental data afford a strong support to the supposition that the meningeal irritation in our patient comprehended such an area as to involve the larger and smaller nerves of the tongue and submaxillary glands in their intra-cranial sites, that probably the excitation was direct and not reflected."

